# Transmission of *Ehrlichia chaffeensis* From an Organ Donor to a Kidney–Pancreas Transplant Recipient

**DOI:** 10.1111/tid.70107

**Published:** 2025-09-18

**Authors:** Praveen Gundelly, Eric Ransom, Zoe Stewart, Brianna Ruch, Arksarapuk Jittirat, Lynn Denny, Jennifer Kasten, Marissa L. Taylor, Johanna S. Salzer, Sridhar V. Basavaraju, Pallavi Annambhotla, David W. McCormick, Arlyn N. Gleaton, Sandor E. Karpathy, Joseph Singleton, Carmen Ramos, Christopher D. Paddock, Laura K. Rothfeldt, Molly Baker, Julian A. Villalba

**Affiliations:** ^1^ University Hospitals Cleveland Health System Cleveland Ohio USA; ^2^ Case Western Reserve University School of Medicine Cleveland Ohio USA; ^3^ Zoonotic Diseases Program Ohio Department of Health Columbus Ohio USA; ^4^ Infectious Diseases Pathology Branch Centers For Disease Control and Prevention Atlanta Georgia USA; ^5^ Rickettsial Zoonoses Branch Centers For Disease Control and Prevention Atlanta Georgia USA; ^6^ Medical Product Safety Branch Division of Healthcare Quality Promotion Centers For Disease Control and Prevention Atlanta Georgia USA; ^7^ Zoonotic Disease Section Arkansas Department of Health Little Rock Arkansas USA; ^8^ Bureau of Communicable Disease Control and Prevention Missouri Department of Health and Senior Services Jefferson City Missouri USA

**Keywords:** donor‐derived infection, *Ehrlichia chaffeensis*, ehrlichiosis, immunocompromised patient, microbial cell‐free DNA metagenomic sequencing, transplant

## 1 Case

A 50‐year‐old man with end‐stage renal disease due to hypertension and diabetes mellitus underwent simultaneous pancreas and left kidney transplantation in July 2024. Immunosuppression included thymoglobulin induction and maintenance with tacrolimus, mycophenolate, and prednisone. Following an uncomplicated recovery, the patient was discharged on Day 10 but readmitted on Day 12 with hypotension. On Day 14, the patient developed a fever (38.8°C). Blood cultures were collected for concern of infection, and the patient received vancomycin and piperacillin‐tazobactam. Computed tomography (CT) scan of the abdomen revealed active extravasation, concerning for pancreatic anastomotic bleed. Transplanted pancreas was surgically removed on Day 15. Antimicrobial therapy was expanded to meropenem and micafungin. No growth was detected using blood cultures. Despite pancreatectomy and broad‐spectrum antimicrobial coverage, the patient remained febrile with progressively worsening leukopenia (0.8 x 10^3^/µL) and thrombocytopenia (23 x 10^3^/µL) on Day 17. A microbial cell‐free DNA (mcfDNA) metagenomic sequencing test (Karius Inc, Redwood City, CA) was performed on plasma collected on Day 17, and doxycycline was initiated. On Day 21, mcfDNA testing detected *Ehrlichia chaffeensis*. His fever resolved within 48 h of starting doxycycline, and he recovered completely by Day 27.

The organ donor was a 27‐year‐old man from Arkansas who died from a traumatic injury. Tick exposure was not ascertained as part of routine organ donation assessment, but the donor's work included outdoor construction. As part of the multi‐agency public health investigation, two additional organ recipients were identified (liver‐right kidney; bilateral lungs), clinicians received treatment guidelines, and specimens were tested from the organ donor and all three recipients at the Centers for Disease Control and Prevention (CDC). Histopathological, serological, and molecular methods were used to evaluate available specimens, including formalin‐fixed paraffin‐embedded (FFPE) tissue blocks [1, 2]. FFPE pancreatic tissue sections were stained by an immunohistochemical method to detect *Ehrlichia* spp. antigens [2].

Serum collected from the index recipient immediately prior to transplant and on Day 31 post‐transplant revealed IgG titers reactive with *E*. *chaffeensis* of < 1:32 and 1:1,024, respectively (at least four‐fold titer change). The liver‐right kidney recipient was admitted with fever and chills on Day 17 post‐transplant. Following notification of *E. chaffeensis* detection by mcfDNA testing in the index recipient, the liver‐right kidney recipient was treated with doxycycline and recovered. The liver‐right kidney recipient's post‐transplant *E. chaffeensis* titer was 1:64 (below the surveillance case definition threshold of ≥ 1:128 for presumptive laboratory evidence), and no molecular detection of *Ehrlichia* resulted from evaluation of blood, serum, or liver biopsy. The bilateral lung recipient was placed on prophylactic doxycycline therapy for 2 weeks, remained asymptomatic, demonstrated a stable titer of < 1:32 pre‐and post‐transplant, and no molecular detection of *Ehrlichia* resulted from serum evaluation.

**FIGURE 1:**
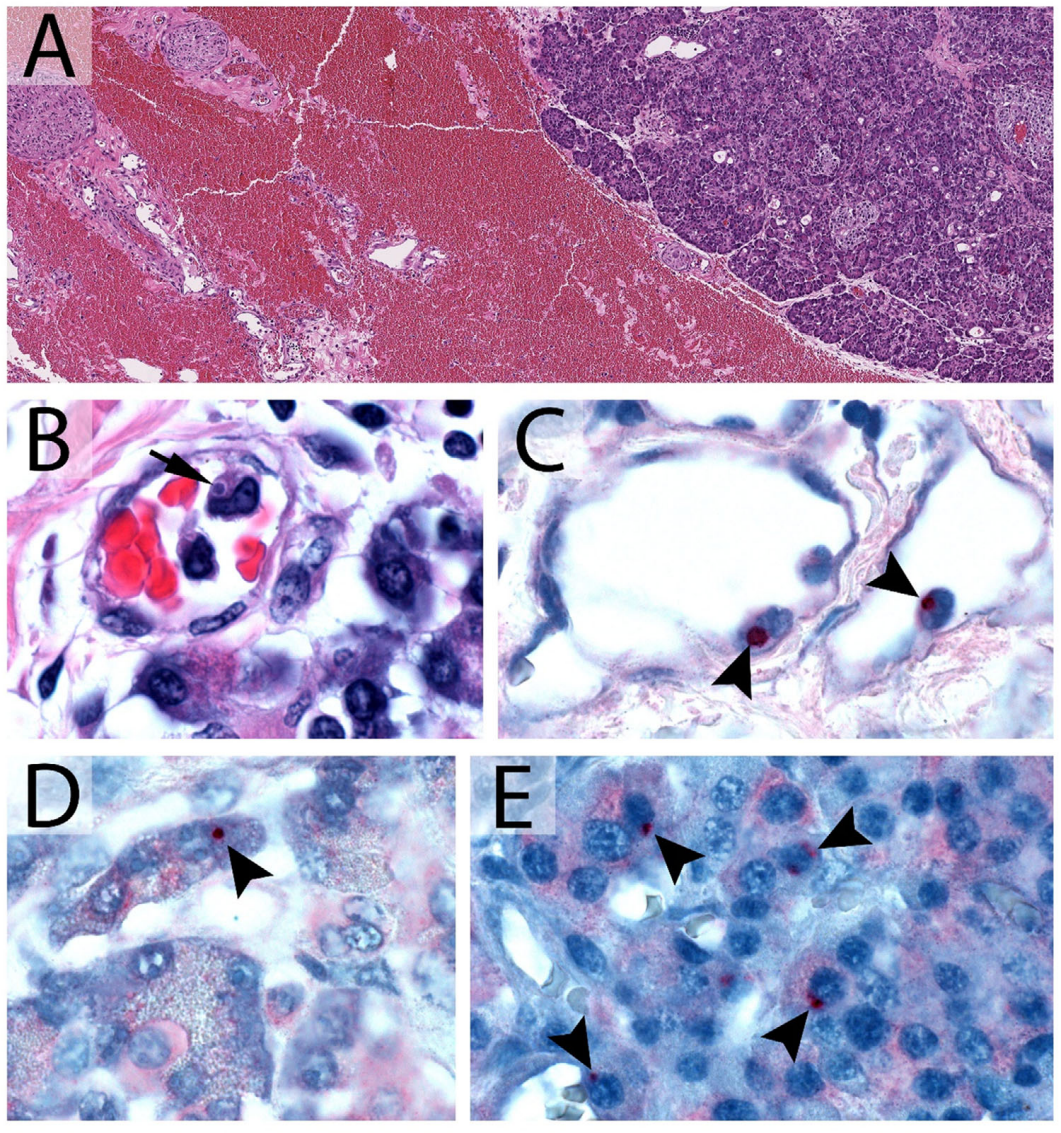
Histopathological and immunohistochemical features of formalin‐fixed, paraffin‐embedded specimens collected from the pancreatic explant procured from the recipient. (A) When stained with hematoxylin and eosin (H&E), the pancreatic explant showed a large dissecting peripancreatic hematoma. (B) *Ehrlichia* morulae (arrow) were seen in H&E‐stained sections by light microscopy in circulating monocytes in the pancreatic and peripancreatic vasculature. (1C‐E) Formalin‐fixed paraffin‐embedded (FFPE) tissue sections were tested by an immunohistochemical method to detect *Ehrlichia* spp. antigens. The immunohistochemical stain highlighted *Ehrlichia* morulae (arrowheads) in (C) intravascular monocytes, (D) pancreatic acinar cells, and (1E) in neuroendocrine cells of the islets of Langerhans. Original magnifications: ×49 (A), ×1000 (B, C, D, E).

Donor plasma and serum collected at time of death were negative for *E. chaffeensis* DNA by real‐time PCR [1], but serology revealed an IgG titer of 1:16,384 to *E. chaffeensis*. Histopathological review of the explanted pancreas revealed a large peripancreatic hemorrhage (Figure 1A), and intracytoplasmic structures compatible with *Ehrlichia* morulae in intravascular mononuclear cells (Figure 1B). Immunohistochemical staining for *E. chaffeensis* highlighted morulae within circulating mononuclear cells (Figure 1C), as well as acinar cells (Figure 1D) and neuroendocrine cells of the pancreatic islets (Figure 1E) [2]. DNA of *E. chaffeensis* was detected by PCR in DNA extracted from sections of the explanted pancreas. Taken together, these findings suggest donor‐derived *E. chaffeensis* infection in the index (pancreas‐left kidney) recipient.

## 2 Discussion

Ehrlichiosis is a tickborne rickettsial disease caused by *E. chaffeensis*, *E. ewingii*, and *E. muris* subsp*. eauclairensis* and treated with doxycycline. *E. chaffeensis* is typically acquired through the bite of an infected lone star tick (*Amblyomma americanum*), with transmission also documented via solid organ transplantation and blood transfusion [3]. Donor‐derived tickborne diseases have been increasingly recognized, often attributed to an unrecognized tick bite in the donor [4, 5, 6]. Collection of tick exposure information during organ donor risk assessments can guide risk characterization and inform clinical management strategies, including empiric treatment and testing for organ recipients. Treatment with doxycycline should be started as soon as ehrlichiosis is suspected, without awaiting test results [7].

Advancements in organ perfusion and preservation allow organs to be widely transported in the United States, and timely diagnosis of donor‐derived infections not endemic to the recipient's region is challenging. In solid organ recipients, mcfDNA metagenomic sequencing can be helpful to identify illness etiology and direct therapy. This method is especially useful for detecting non‐culturable organisms or infections with prolonged incubation, including *E. chaffeensis* [8].

Cases of *E. chaffeensis* infection have been reported in solid organ recipients as both donor‐derived infections and acquired by tick bite post‐transplant. Cases are typically more severe due to the patients’ immunosuppression [3]. Although *E. chaffeensis* is more commonly associated with kidney and liver transplants [3], at least three cases of ehrlichiosis or anaplasmosis have been documented in pancreas or kidney–pancreas recipients [9]. The present case is unique in demonstrating *E. chaffeensis* tropism for pancreatic islet and acinar cells by tissue‐based techniques.

## Author Contribution

**Praveen Gundelly**: writing – review and editing. **Eric Ransom**: writing – review and editing. **Zoe Stewart**: writing – review and editing. **Brianna Ruch**: writing – review and editing. **Arksarapuk Jittirat**: writing – review and editing. **Lynn Denny**: writing – review and editing. **Jennifer Kasten**: writing – review and editing. **Marissa L. Taylor**: writing – review and editing. **Johanna S. Salzer**: writing – review and editing. **Sridhar V. Basavaraju**: writing – review and editing. **Pallavi Annambhotla**: writing – review and editing. **David W. McCormick**: writing – review and editing. **Arlyn N. Gleaton**: writing – review and editing. **Sandor E. Karpathy**: writing – review and editing. **Joseph Singleton**: writing – review and editing. **Carmen Ramos**: writing – review and editing. **Christopher D. Paddock**: writing – review and editing. **Laura K. Rothfeldt**: writing – review and editing. **Molly Baker**: writing – review and editing. **Julian A. Villalba**: writing – review and editing.

## Disclosure

The findings and conclusions in this report are those of the authors and do not necessarily represent the official position of the Centers for Disease Control and Prevention or Arkansas Department of Health.

## Conflicts of Interest

The authors declare no conflicts of interest.

## Data Availability

The data in this study are collected and managed by the US CDC; however, the corresponding author had full access to all the data in the study and had the final responsibility for the decision to submit for publication.
